# Low Vision Rehabilitation Service Utilization Before and After Implementation of a Clinical Decision Support System in Ophthalmology

**DOI:** 10.1001/jamanetworkopen.2022.54006

**Published:** 2023-02-03

**Authors:** Xinxing Guo, Michael V. Boland, Bonnie K. Swenor, Judith E. Goldstein

**Affiliations:** 1 Wilmer Eye Institute, Johns Hopkins University School of Medicine, Baltimore, Maryland; 2 Department of Ophthalmology, Massachusetts Eye and Ear and Harvard Medical School, Boston; 3 Cochlear Center for Hearing and Public Health, Department of Epidemiology, Johns Hopkins Bloomberg School of Public Health, Baltimore, Maryland; 4 Disability Health Research Center, Johns Hopkins University, Baltimore, Maryland; 5 Johns Hopkins University School of Nursing, Baltimore, Maryland

## Abstract

**Importance:**

Electronic clinical decision support systems apply clinical guidelines in real time and offer a new approach to improve referral and utilization of low vision rehabilitation (LVR) care.

**Objective:**

To characterize patients and factors associated with LVR service utilization with and without the use of an electronic health record (EHR) clinical decision support system (CDSS) alert.

**Design, Setting, and Participants:**

Quality improvement study using EHR data to compare patients who did and did not utilize LVR service after referral between November 6, 2017, and October 5, 2019, (primary) and to assess overall service utilization rate from September 1, 2016, to April 2, 2021, regardless of referral status (secondary). Participants in the primary analysis were patients at a large ophthalmology department in an academic medical center in the US who received an LVR referral recommendation from their ophthalmologist according to the CDSS alert. The secondary analysis included patients with best documented visual acuity (BDVA) worse than 20/40 before, during, and after the CDSS implementation. Data were analyzed from August 2021 to April 2022.

**Exposures:**

Number and locations of referral recommendations for LVR service according to the CDSS alert in the primary analysis; active CDSS implementation in the secondary analysis.

**Main Outcomes and Measures:**

LVR service utilization rate was defined as the number of patients who accessed service among those who were referred (primary) and among those with BDVA worse than 20/40 (secondary). EHR data on patient demographics (age, sex, race, ethnicity) and ophthalmology encounter characteristics (numbers of referral recommendations, encounter location, and BDVA) were extracted.

**Results:**

Of the 429 patients (median [IQR] age, 71 [53 to 83] years; 233 female [54%]) who received a CDSS-based referral recommendation, 184 (42.9%) utilized LVR service. Compared with nonusers of LVR, users were more likely to have received at least 2 referral recommendations (12.5% vs 6.1%; χ^2^_1_ = 5.29; *P* = .02) and at an ophthalmology location with onsite LVR service (87.5% vs 78.0%; χ^2^_1_ = 6.50; *P* = .01). Onsite LVR service (odds ratio, 2.06; 95% CI, 1.18-3.61) persisted as the only statistically significant factor after adjusting for patient demographics and other referral characteristics. Among patients whose BDVA was worse than 20/40 before, during, and after the CDSS implementation regardless of referral status, the LVR service utilization rate was 6.1%, 13.8%, and 7.5%, respectively.

**Conclusions and Relevance:**

In this quality improvement study, ophthalmologist referral recommendations and onsite LVR services at the location where patients receive other ophthalmic care were significantly associated with service utilization. Ophthalmology CDSSs are promising tools to apply clinical guidelines in real time to improve connection to care.

## Key Points

**Question:**

How was an ophthalmology clinical decision support system (CDSS) associated with low vision service utilization?

**Findings:**

In this quality improvement study including 429 patients, service utilization was found in 42.9% of the patients who received a referral recommendation during the electronic health record–related CDSS active phase and was associated with onsite service provision. The service utilization rate in patients with worse than 20/40 visual acuity was the highest when the CDSS alert was active.

**Meaning:**

These findings suggest that implementing a CDSS in eye care coupled with onsite service provision may be useful in applying clinical guidelines to improve utilization of low vision care.

## Teaser

This quality improvement study evaluates low vision rehabilitation service use before and after implementation of an electronic health record–based clinical decision support system at a large ophthalmology department in an academic medical center.

## Introduction

Fewer than 10% of people in need of low vision rehabilitation (LVR) care utilize the service.^1^ Organizational efforts by the American Academy of Ophthalmology (AAO), the American Optometric Association, and other entities in the US continue to make efforts to improve the delivery of LVR services with a focus on connecting patients to care.^2,3,4,5^ Due to a lack of LVR service awareness by the general population, the outpatient US care delivery models typically follow the pipeline of (1) patient identification by the ophthalmologist or optometrist, (2) recommendation and referral to LVR service by the clinician, and (3) service utilization by the patient.^6,7,8^ To properly evaluate the efficiency of the pipeline and to better address the gaps in care delivery, reliable, measurable, and sustainable outcome metrics are needed. However, data regarding LVR referral are limited and information on service utilization after referral is mostly unknown.^9,10^ Only with systematic approaches and a reliable audit trail can LVR service utilization determinants be appropriately assessed and improvements throughout the pipeline of care be implemented.

In our previous work, we developed and tested an electronic health record (EHR)-based ophthalmology clinical decision support system (CDSS) that follows the pipeline by identifying patients who may benefit from LVR services. When referral criteria were met, ophthalmologists received an EHR-generated alert and documented their response to the LVR referral recommendation. We previously reported on the development of the CDSS^11^ and the ophthalmologist referral patterns, which revealed nearly 15% of patients meeting visual acuity or neurologic-related visual field diagnosis criteria were referred.^12^

In this work, we rely on the same sample of patients as reported previously.^12^ However, only the subset meeting the visual acuity criteria were included. For the primary analysis, we compared the characteristics of patients who did and did not utilize LVR services after receiving a referral recommendation from their ophthalmologists according to the CDSS alert and identified factors associated with service utilization. We hypothesized that patients who received more than 1 referral recommendation and those who sought eye care with onsite LVR service were more likely to utilize care.^13^ In the secondary analysis, we assessed the LVR service utilization rate in relation to the CDSS implementation, with the hypothesis that service utilization rate would be higher during the CDSS active phase as compared with before or after.

## Methods

### Study Setting and Participants

The primary analysis was part of a quality improvement project at the Johns Hopkins Wilmer Eye Institute aiming to develop and test an EHR-based CDSS for identifying patients who might benefit from LVR services between November 6, 2017 and April 5, 2019.^11^ The Johns Hopkins School of Medicine institutional review board determined that the project was exempt from review. Therefore, an informed consent was not applicable to the project. This study followed the Standards for Quality Improvement Reporting Excellence (SQUIRE) and Strengthening the Reporting of Observational Studies in Epidemiology (STROBE) reporting guidelines.

A convenience sample of 15 ophthalmologists from 8 ophthalmology subspecialties participated in the project. The mandatory CDSS alert appeared and recorded the ophthalmologist’s response when a patient met the alert criteria. These criteria were designed to reflect the AAO guidelines for LVR referral and included best-documented visual acuity (BDVA) worse than 20/40 in the better eye or a diagnosis related to hemianopia or quadrantanopia (*International Statistical Classification of Diseases and Related Health Problems, Tenth Revision* codes of H53.47 or H63.46). The alert was suppressed when a patient: (1) was younger than 5 years old, (2) had an ophthalmic surgery scheduled in the next 3 months or performed in the past 3 months, (3) had an LVR clinic visit in the past 12 months, or (4) had prior CDSS action(s) that suppressed the alert activation for the current encounter. Available responses for the ophthalmologists included “order” and “don’t order” for LVR referral. No additional intervention other than the usual care practices were followed regarding connecting patients to LVR service.^12^ Although the previous report examined factors associated with LVR referral on the encounter level,^12^ the current analysis was conducted on the patient level to determine factors associated with LVR service utilization. Patients were included in the primary analysis if they had at least 1 encounter where their ophthalmologist recommended LVR referral by responding “order referral” to the alert according to the BDVA criteria only.

### Low Vision Rehabilitation Service Utilization

To ensure adequate time for patients to utilize LVR service, each patient was followed through October 5, 2019 with a 6-month minimum follow-up period. A patient was considered to have utilized LVR service if they completed an LVR clinic visit at the study institution between their first encounter where an ophthalmologist recommended LVR referral and October 5, 2019.

### Electronic Health Record Data Extractions

Data on patient demographics including age, sex, self-reported race and ethnicity from an EHR predefined list according to the federal government definition, and encounter characteristics including numbers of encounters with a referral recommendation, referral encounter location, and BDVA were extracted from the EHR. Race and ethnicity were assessed to better understand the patient population that did and did not utilize LVR service. The 6 ophthalmologist referral locations were categorized into those with onsite LVR service (3 locations) and those without LVR service (3 locations). BDVA was categorized into (1) worse than 20/40 and 20/60 or better, (2) worse than 20/60 and better than 20/200, (3) 20/200 or worse and better than 20/500, and (4) 20/500 or worse.

### Evaluation of Service Utilization in Relation to CDSS Implementation

A secondary evaluation was conducted to determine the differences in LVR service utilization rate before (September 2016 to March 2017), during (November 2017 to April 2019), and after (May 2020 to April 2021) the CDSS implementation among 12 of the 15 participating ophthalmologists. The CDSS alert was inactive before and after the implementation period. To enable comparison of the service utilization rate among patients who would have met CDSS criteria between active and inactive alert periods, patients were classified as having utilized LVR services when BDVA was worse than 20/40 and a subsequent encounter with an LVR clinician occurred within 6 months regardless of whether a referral recommendation was placed. Clinical and service utilization data from the 3 periods were extracted from the EHR. Patients were defined as eligible if they had available BDVA data and did not meet any of the aforementioned alert suppression criteria. Patients who had at least 1 encounter where BDVA was worse than 20/40 in the better eye were considered as meeting the CDSS criteria.

### Statistical Analyses

For the primary analysis, demographic and clinical characteristics between patients that did and did not utilize LVR service were compared using the Wilcoxon Rank Sum Test for age, and χ^2^ tests for other variables. Factors associated with LVR service utilization for patients who received LVR referral recommendations using BDVA of worse than 20/40 criteria only were assessed using a multivariable logistic regression model adjusting for patient age, sex, race (categorized as Black or African American, Asian, White, Others [including American Indian or Alaska Native, Native Hawaiian, Other, Other Pacific Islander, and Choose Not to Disclose]), ethnicity (Hispanic or Latino, Not Hispanic or Latino), number of referral recommendations, the presence or absence of onsite LVR service at the ophthalmologist’s referring location, and BDVA categories. Patients who had a diagnosis associated with neurological visual field defects (ie, hemianopia or quadrantanopia) were excluded because the diagnosis criterion was introduced 7 months after the CDSS implementation,^11^ and referral rates in this group of patients were significantly higher as compared with those with visual acuity loss alone. We hypothesized that the acute nature of the neurologic-related visual field loss coupled with the lack of treatment options may be a source of the differing rates.^12^ LVR is commonly deferred when individuals are under active medical or surgical therapy in anticipation of some recovery of vision. To explore whether ophthalmologists with a higher patient referral rate had a higher patient service utilization rate, we examined the Pearson correlation coefficient between patient LVR service utilization rate and patient referral rate on the ophthalmologist level in the 12 ophthalmologists who referred at least 10 patients during the study period. We chose the 10-patient referral threshold for a more reliable estimate of patient service utilization rates on the individual physician level. Referral rate was calculated as the number of patients where their ophthalmologist responded “order referral” divided by the total number of their patients with at least 1 encounter where BDVA was worse than 20/40.

For the secondary analysis, we compared institutional patient LVR service utilization before, during, and after the CDSS implementation among the patients whose BDVA was worse than 20/40 in the better eye and did not meet any of the alert suppression criteria using the χ^2^ test. To enable comparison, the service utilization rate was determined among patients who would have met the CDSS criteria, rather than among patients in whom an alert appeared and referral was recommended.

All statistical analyses were conducted using Stata/SE 17 (Stata Corp., USA). Statistical significance was set at *P* < .05, and 2-sided values were presented. Data were analyzed from August 2021 to April 2022.

## Results

### Factors Associated With LVR Service Utilization

Overall, 429 patients (median [IQR] age: 71 [53 to 83] years, 233 female [54%]) received at least 1 LVR referral recommendation from the 15 participating ophthalmologists over 17 months when they had BDVA worse than 20/40. Among them, 145 (33.8%) had at least 20/60 BDVA, 160 (37.3%) had BDVA between 20/60 and 20/200, and 184 (42.9%) utilized LVR service. (Table 1) The median (IQR) time for patients to utilize LVR after the referral recommendation was 73 (28 to 129) days.

**Table 1.** Patient Characteristics by LVR Service Utilization Status

Characteristic	Descriptive analysis (n = 429)			Regression analysis outcome: LVR service utilized,^a^ Odds Ratio (95% CI)
	LVR Service, No. (%)		*P* value	
	Utilized (n = 184)	Not utilized (n = 245)		
Age, median (25th, 75th percentile), y	72 (55, 83)	71 (51, 82)	.56	NA
Age Groups, y				
≥5 to <20	11 (6.0)	16 (6.5)	.98	1.06 (0.36-3.13)
≥20 to <40	16 (8.7)	25 (10.2)		1 [Reference]
≥40 to <65	44 (23.9)	58 (23.7)		1.36 (0.62-2.99)
≥65 to <80	50 (27.2)	66 (26.9)		1.26 (0.59-2.73)
≥80	63 (34.2)	80 (32.7)		1.35 (0.63-2.89)
Sex				
Female	96 (52.2)	137 (55.9)	.44	1 [Reference]
Male	88 (47.8)	108 (44.1)		1.23 (0.81-1.85)
Race^b^				
Asian	11 (6.0)	11 (4.6)	.91	1.39 (0.54-3.60)
Black or African American	61 (33.5)	83 (34.4)		0.96 (0.61-1.52)
White	97 (53.3)	131 (54.4)		1 [Reference]
Others^c^	13 (7.1)	16 (6.6)		0.97 (0.38-2.44)
Ethnicity^b^				
Non-Hispanic	166 (94.3)	232 (97.9)		1 [Reference]
Hispanic	10 (5.7)	5 (2.1)		2.72 (0.78-9.47)
No. of referral recommendations				
1	161 (87.5)	230 (93.9)	.02^d^	1 [Reference]
≥2	23 (12.5)	15 (6.1)		1.96 (0.97-3.98)
Referral service location				
Location without onsite LVR service	23 (12.5)	54 (22.0)	.01^d^	1 [Reference]
Location with onsite LVR service	161 (87.5)	191 (78.0)		2.06 (1.18-3.61)^d^
BDVA Category				
<20/40, ≥20/60	65 (35.3)	80 (32.7)	.47	1 [Reference]
<20/60, >20/200	72 (39.1)	88 (35.9)		0.93 (0.53-1.51)
≤20/200, >20/500	20 (10.9)	39 (15.9)		0.65 (0.33-1.26)
≤20/500	27 (14.7)	38 (15.5)		1.00 (0.53-1.88)

Abbreviations: BDVA, best-documented visual acuity; LVR, low vision rehabilitation.

^a^ Regression analysis conducted in patients with complete covariate data (408 of 429 [95%]), adjusting for age, sex, race, ethnicity, number of referrals, whether there was onsite LVR service at the referral clinic, and BDVA categories.

^b^ Race and ethnicity information missing for 6 (1.4%) and 16 (3.7%) patients, respectively.

^c^ Others included American Indian or Alaska Native, Native Hawaiian, Other, Other Pacific Islander, and Choose Not to Disclose.

^d^ Statistically significant at 2-sided *P* < .05 level.

Compared with patients who did not utilize LVR, those who did were more likely to have received at least 2 referral recommendations (12.5% vs 6.1%; χ^2^_1_ = 5.29; *P* = .02) and were more likely to have received referral recommendations from clinic locations with onsite LVR service (87.5% vs 78.0%; χ^2^_1_ = 6.50; *P* = .01). No differences were found regarding patient age, sex, race and ethnicity, or BDVA category by service utilization. In the multivariable regression model, clinic locations with onsite LVR services remained the only statistically significant factor associated with service utilization (odds ratio, 2.06; 95% CI: 1.18 to 3.61; *P* = .01) after adjusting for patient demographics and other referral characteristics (Table 1).

Of the 15 participating ophthalmologists, referral rates for their patients with worse than 20/40 BDVA ranged between 1.2% and 33.8% with a median of 15.6%; patient LVR service utilization rates ranged between 14.3% and 100% with a median of 43.2% (eTable in Supplement 1). We evaluated the patient LVR utilization rate by ophthalmologist referral rate (Figure). In the 12 physicians who made referral recommendations to at least 10 patients, we observed a Pearson correlation coefficient of 0.27 (95% CI: −0.36 to 0.73) between LVR utilization rate and referral rate.

**Figure.**
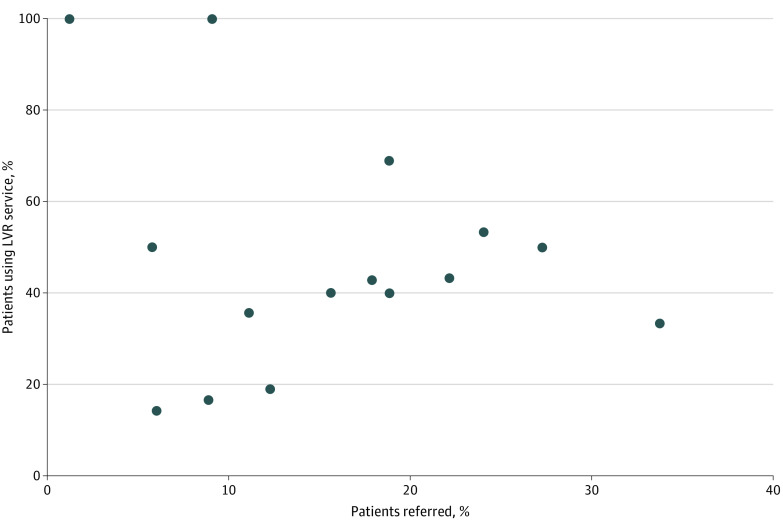
Patient Referral Rate and Patient LVR Service Utilization Rate by Ophthalmologist Referral rate is calculated as the number of patients where the ophthalmologist responded “order referral” divided by the total number of that ophthalmologist’s patients with at least 1 encounter where the CDSS alert appeared. Patient LVR service utilization rate is calculated as the number of patients with an LVR clinic visit at the same institute after the ophthalmologist responded “order referral” within 6 months.

### Service Utilization Before, During, and After CDSS Implementation

To assess the potential impact of the CDSS on LVR utilization, we compared institutional LVR utilization rates among patients who would have met the CDSS visual acuity criteria (BDVA worse than 20/40) regardless of referral status before, during, and after the alert mandate. This separate analysis was conducted using patients of the 12 ophthalmologists who provided care in the same institute from September 1, 2016 to April 2, 2021. The proportion of patients with BDVA worse than 20/40 among those eligible for these 3 phases differed statistically: 10.8% before, 11.3% during, and 9.6% after the active CDSS alert phase (χ^2^_2_ = 43.09; *P* < .001). Among them, the LVR service utilization rate was 6.1% before, 13.8% during, and 7.5% after the CDSS active alert regardless of whether a referral was recommended (χ^2^_2_ = 60.01; *P* < .001) (Table 2). Specifically, of the 2227 patients who had BDVA worse than 20/40 while the alert was active, the LVR service utilization rate was significantly higher among the 387 patients who received a referral recommendation than the 1840 patients who did not (41.6% vs 8.0%; χ^2^_1_ = 303.14; *P* < .001).

**Table 2.** LVR Service Utilization Rate Before, During, and After Electronic CDSS Alert Period^a^

Parameters	CDSS Alert mandate		
	Before (September 1, 2016 to March 31, 2017)	During (November 6, 2017 to April 5, 2019)	After (May 1, 2020 to April 2, 2021)
CDSS alert status	Inactive	Active	Inactive
Clinic days, No.	155	352	226
Eligible patients,^b^ No.	9477	19 728	13 153
Patients meeting alert visual acuity criteria,^c^ No. (%)^d^	1025 (10.8)	2227 (11.3)	1258 (9.6)
Patients utilized LVR service,^e^ No. (%)^d^	63 (6.1)	308 (13.8)	94 (7.5)

Abbreviations: CDSS, clinical decision support system; LVR, low vision rehabilitation.

^a^ Summary data represented for 12 of the 15 participating physicians who worked at the institute from September 1, 2016, to April 2, 2021.

^b^ Eligible patients defined as patients whose encounters had available visual acuity data and did not meet any of the alert suppression criteria (ie, younger than 5 years old, had ophthalmic surgery scheduled in the next 3 months or performed in the past 3 months, had prior LVR clinic visit[s] within the past 12 months).

^c^ Patients who had at least 1 encounter during the respective period where best documented visual acuity was worse than 20/40 in the better eye. Percentage calculated as the number of patients meeting alert visual acuity criteria by all eligible patients.

^d^ Statistically significant at 2-sided *P* < .001 level between the 3 periods.

^e^ Patients who accessed LVR services within 6 months of their last encounter where CDSS visual acuity criteria were met. For patients during active CDSS period, the number included those whose ophthalmologist responded “order” and “don’t order” to the alert. Percentage calculated as the number of patients who took up service by the number of patients meeting alert visual acuity criteria.

## Discussion

Ophthalmologist referral recommendations are critical in determining whether patients utilize LVR services. Patients who may benefit from LVR are largely unaware or may hold misconceptions of the service until their ophthalmologist, optometrist, neurologist, or other clinician introduces the idea and recommends a referral.^7,14,15,16^ Thus, clinician endorsement and advocacy play an essential role in LVR service delivery. Among patients who received a referral recommendation, 2 out of 5 utilized LVR services, and notably worsening visual acuity was not associated with LVR utilization. Having onsite LVR services at clinics where patients obtained their ophthalmic care increased LVR utilization. Our findings highlight that EHR-based CDSSs provide a viable mechanism to systematically aid LVR referral and improve service utilization.

### Factors Associated With Low Vision Rehabilitation Service Utilization

Study findings provide insight into optimal strategies to incorporate LVR delivery, specifically as it relates to service delivery location and the significance of the referral by the ophthalmologist. LVR services offered at the same site of the referring ophthalmologist were associated with increased utilization, most likely due to improved convenience and accessibility for the patient and service familiarity by the ophthalmologist. LVR service utilization was significantly higher in patients who received referral recommendations than their counterparts who did not during the CDSS active period. Ophthalmologists with higher referral rates may have higher patient LVR utilization, although assessment was limited by the 12 physicians with at least 10 patients referred. We hypothesize that the positive correlation, albeit weak, may be a result of a familiarity and endorsement of the service translating to patient adherence to the referral recommendation. Among the patients who received LVR referral recommendations, the number of referral recommendations differed between service utilizers and nonutilizers, with twice the proportion of utilizers receiving at least 2 referral recommendations compared with nonutilizers. However, the association did not persist after adjusting for patient demographics and other referral characteristics. This may be due to the relatively small number of patients who received at least 2 referral recommendations. A higher number of referral recommendations for a given patient may reflect more severe impairment, persistent or worsening patient concerns, or a need for reinforcement in patients who may be forgetful or focused on restoration treatments. Because of the discussion required, time constraints within an ophthalmology visit, and the implications of the referral (eg, permanent vision loss), patients may require repeated conversations with their ophthalmologists before taking action and utilizing LVR services.^17^ As acceptance and adaptation to vision loss is individual and the need for LVR may depend on a change in family or social support, CDSSs may serve as a needed reminder and provide a mechanism for patients who may be lost to follow up LVR care.

### Utility of Clinical Decision Support System

Previous reports evaluating LVR referral and service utilization largely relied on survey data and manual medical record review,^9,10,17,18^ which was inefficient, labor-intensive, and error-prone.^19^ Using the EHR-based CDSS with visual acuity criteria similar to AAO guidelines (while excluding patients who were less likely to be in need of services and absent of additional intervention), we found an LVR service utilization rate of 42.9% for patients who received a referral recommendation. There are limited comparative data available as the few reports on LVR utilization were outside the US health system, relied on patient self-report, or used different visual acuity referral criteria. In these reports, utilization of LVR service ranged between 49% and 97%.^7,17,18,20^

Across medical specialties, CDSSs have been shown to be effective at improving health care delivery processes and outcomes.^21,22,23,24^ Endocrinology, primary care, radiology, and other specialties have initiated CDSS and associated quality improvement efforts, but to date, despite preliminary interest,^25^ there are no published works that we know of in ophthalmology examining the use of CDSSs. In fairly high-volume ophthalmology clinics, the CDSS not only provided a reliable tracking mechanism for auditing service referral and utilization, but also served as a practice guideline reminder to ophthalmologists. Although not well studied, CDSSs may also serve to minimize unintended physician biases.^26^ In the secondary analysis which includes 12 out of the 15 ophthalmologists, patients who did not receive a referral recommendation during the CDSS active period had a similar LVR utilization rate (8.0%) when compared with the CDSS inactive period (6.1% before and 7.5% after alert mandate). This contrasts to the LVR utilization rate (41.6%) in patients who received referral recommendations during the active phase, highlighting the vital role of ophthalmologists in driving LVR utilization. As 85% of the participating ophthalmologist users found the alert to be useful in identifying candidates for referral,^12^ and given that utilization of LVR was significantly less when the alert was inactive, implementing CDSS in ophthalmology may offer a new and sustainable approach to meeting clinical guidelines and standardizing LVR delivery practices. Without the implementation of a CDSS, there are no consistent or sustainable systems to track ophthalmologist LVR referrals and patient service utilization. Given this, baseline LVR utilization rates have remained mostly unknown and ongoing quality improvement interventions are difficult to measure. The CDSS in this this work enables comparison of service utilization using clinical data obtained before, during, and after the CDSS mandate.

### Limitations

This study has limitations. Additional factors not explored that may have impacted LVR service utilization rates include the COVID-19 pandemic, LVR appointment wait times and availability, transportation, insurance status, and other barriers to accessing care. Referral criteria outlined in the AAO guidelines^2^ such as loss of visual ability, contrast sensitivity, or visual field were not included when generating an alert due to EHR data limitations, documentation practices, and participating ophthalmologist’s preferences. A larger study sample size is needed to further evaluate the reported associations. Future approaches should incorporate other referral sources (optometrists, neurologists, and so forth), and a more comprehensive definition of low vision. However, by their very nature and design, CDSSs should be user-centered. Thus it may be necessary to set different criteria according to physician and patient preferences. Interventions overlaying the CDSS provide additional research opportunities to improve eye care delivery with measurable outcomes.

### Conclusion

In this quality improvement study, patients were more apt to utilize LVR services after receiving a referral recommendation by an ophthalmologist and when LVR services were provided at the same location where they received other ophthalmic care. Ophthalmology CDSSs are promising tools to apply clinical guidelines in real-time, automate quality assurance, and improve utilization of LVR care.

## Notes

Supplement 1. **eTable. **Referral Rate and Patient LVR Utilization Rate Among Patients Who Had BDVA Less Than 20/40 by Physician

Supplement 2.
Data Sharing Statement
