# Supplementary material for: Low Vision Rehabilitation Service Utilization Before and After Implementation of a Clinical Decision Support System in Ophthalmology
Source: JAMA Netw Open. 2023 Feb 3;6(2):e2254006. doi: 10.1001/jamanetworkopen.2022.54006 (PMC9898817; doi:10.1001/jamanetworkopen.2022.54006)
Supplement: Supplement 1. — eTable. Referral Rate and Patient LVR Utilization Rate Among Patients Who Had BDVA Less Than 20/40 by Physician [file jamanetwopen-e2254006-s001.pdf]

Supplemental Online Content

Guo X, Boland MV, Swenor BK, Goldstein JE. Low vision rehabilitation service utilization before and after implementation of a clinical decision support system in ophthalmology. *JAMA Netw Open*. 2023;6(2):e2254006. doi:10.1001/jamanetworkopen.2022.54006

eTable. Referral Rate and Patient LVR Utilization Rate Among Patients Who Had BDVA Less Than 20/40 by Physician

This supplemental material has been provided by the authors to give readers additional information about their work.

© 2023 Guo X et al. *JAMA Network Open.*

eTable 1. Referral Rate and Patient LVR Utilization Rate Among Patients Who Had BDVA Less Than 20/40 by Physician

	BDVA<20/40	Referral Recommended			LVR Service Utilization	
	n	n	% [a]		n	% [b]
Comprehensive Ophthalmology Physician 1	167	37	22.2		16	43.2
Comprehensive Ophthalmology Physician 2	116	7	6.0		1	14.3
Cornea Physician 1	171	21	12.3		4	19.0
Cornea Physician 2	126	14	11.1		5	54.8
Glaucoma Physician 1	88	8	9.1		8	100.0
Glaucoma Physician 2	198	54	27.3		27	50.0
Glaucoma Physician 3	311	105	33.8		35	33.3
Neuro Ophthalmology Physician 1	187	45	24.1		24	53.3
Neuro Ophthalmology Physician 2	154	29	18.8		20	69.0
Oculoplastics Physician 1	96	15	15.6		6	40.0
Pediatrics & Adult Strabismus Physician 1	53	10	18.9		4	40.0
Pediatrics & Adult Strabismus Physician 2	242	14	5.8		7	50.0
Retina Physician 1	244	3	1.2		3	100.0
Retina Physician 2	352	63	17.9		27	42.9
Uveitis Physician 1	135	12	8.9		2	16.7
Total	2530	429	17.0		184	42.9

LVR: low vision rehabilitation; BDVA: best-documented visual acuity

[a] Patient referral rate was calculated as the number of patients where the physician responded “order referral” divided by the total number of patients with at least 1 encounter where BDVA was worse than 20/40.

[b] Patient LVR service utilization rate was calculated as the number of patients who completed an LVR clinic visit between their first encounter where their physician responded “order referral” and October 5th, 2019 divided by the total number of patients who received referral recommendations from the physician.

© 2023 Guo X et al. *JAMA Network Open.*
