# Supplementary material for: Low Vision Rehabilitation Service Utilization Before and After Implementation of a Clinical Decision Support System in Ophthalmology
Source: JAMA Netw Open. 2023 Feb 3;6(2):e2254006. doi: 10.1001/jamanetworkopen.2022.54006 (PMC9898817; doi:10.1001/jamanetworkopen.2022.54006)
Supplement: Supplement 2. — Data Sharing Statement [file jamanetwopen-e2254006-s002.pdf]

Data Sharing Statement

Guo. Low Vision Rehabilitation Service Utilization Before and After Implementation of a Clinical Decision Support System in Ophthalmology. *JAMA Netw Open*. Published February 03, 2023. doi:10.1001/jamanetworkopen.2022.54006

Data
Data available: No
